# Selection history: How reward modulates selectivity of visual attention

**DOI:** 10.3758/s13423-017-1380-y

**Published:** 2017-10-04

**Authors:** Michel Failing, Jan Theeuwes

**Affiliations:** Department of Experimental and Applied Psychology, Vrije Universiteit Amsterdam, 1081 BT Amsterdam, The Netherlands

**Keywords:** Attention, Attentional capture, Visual selective attention, Priming

## Abstract

Visual attention enables us to selectively prioritize or suppress information in the environment. Prominent models concerned with the control of visual attention differentiate between goal-directed, top-down and stimulus-driven, bottom-up control, with the former determined by current selection goals and the latter determined by physical salience. In the current review, we discuss recent studies that demonstrate that attentional selection does not need to be the result of top-down or bottom-up processing but, instead, is often driven by lingering biases due to the “history” of former attention deployments. This review mainly focuses on reward-based history effects; yet other types of history effects such as (intertrial) priming, statistical learning and affective conditioning are also discussed. We argue that evidence from behavioral, eye-movement and neuroimaging studies supports the idea that selection history modulates the topographical landscape of spatial “priority” maps, such that attention is biased toward locations having the highest activation on this map.

In everyday life, visual input is used to guide our behavior. We intentionally search for our bag on the luggage carousel at the airport while keeping in mind its shape and color to facilitate search. This template makes it easier to find our bag among the many distracting similarly looking bags. When searching with a goal for particular objects, we may sometimes experience that we attend to things in our environment for which we had no intention to look for. We may inadvertently attend to the waving hand of our friend who already found his bag or the flashing light next to yet another luggage carousel that is about to start moving.

The question for how we search the environment, and more generally how we parse information from the environment, is studied in the context of attentional control. All models of selective attention have described attentional control as the result of the above described interplay between voluntary, top-down, or goal-driven control and automatic, bottom-up, or stimulus driven control (e.g., Corbetta & Shulman, 2002; Itti & Koch, 2001; Theeuwes, 2010). In a recent paper, Awh, Belopolsky, and Theeuwes (2012) pointed out that this classic theoretical dichotomy may no longer hold as there is a significant explanatory gap: Several selection biases can neither be explained by current selection goals nor by the physical salience of potential targets. Awh et al. suggested that a third category labelled as selection history competes for selection. This category describes lingering selection biases formed by the history of attentional deployments that are unrelated to top-down goals or the physical salience of items.

The current review takes the previous paper of Awh et al. (2012) further and discusses recent findings of key behavioral, eye-movement, and neuroimaging studies that demonstrate effects that should be considered to be the result of selection history (i.e., lingering selection biases based on history). While focusing on reward-based history effects, other history effects such as (intertrial) priming, affective conditioning and statistical learning are also discussed. In the following sections, we will first briefly introduce some of the critical concepts around (selective) attention and review the recent surge in literature investigating how attention is affected by reward (history) as well as other types of selection history, before we embed the findings into the bigger picture of how selection history contributes to the understanding of the world around us.

## Top-down and bottom-up attentional control

Irrespective of whether we search the environment covertly or overtly, classic studies have isolated two categories of processes demarking how visual attentional selection is controlled. Thinking about the example of how you search for your bag at the airport, one intuitive way to categorize attentional control is in terms of one’s own goals, intentions, or beliefs. Indeed, it is obvious that attending certain cues (e.g., the screens above the luggage carousels indicating which flight’s luggage is about to be unloaded) might help to achieve your goal of finding your bag, while attending others may not. This so-called top-down (also called endogenous or goal-directed) attentional control is thought to be volitional and under the control of the observer at any given point in time (Posner, 1980; Treisman & Gelade, 1980; Theeuwes, 2010; Wolfe, 2007; Yantis, 2000). Since top-down processes are under volitional control, the observer has to intentionally activate them, which usually takes a considerable amount of time and effort.

Searching for your bag, however, is not a simple task when the airport is very busy and time to search is limited. The flashing light of the other luggage carousel or your waving friend might also attract your attention even though you were not actively searching for any of them. These examples of bottom-up (also called exogenous or stimulus-driven) control highlight that selection is also determined by the properties of the environment. In particular, physical salience of a stimulus relative to its immediate surroundings determines how likely it is that this stimulus “grabs,” or captures, attention. The salience of a stimulus is usually defined in terms of low-level stimulus features such as luminance, orientation, color, or motion (e.g., Theeuwes, 1991a, 1992, 1994; Itti & Koch, 2001). Contrary to top-down attentional control, bottom-up control is thought to occur involuntarily (i.e., independent of one’s goals and intentions) but also far more quickly and effortlessly. Findings from studies on bottom-up attentional control are commonly interpreted in terms of a physically salient event or stimulus drawing attention on its own.

## Priority maps: An integration of top-down and bottom-up processes

The dichotomy between top-down and bottom-up attentional control has sparked a great deal of research investigating its explanatory power by pitting the respective influence of both types of control against each other. For example, research demonstrated that when searching for a given target, physically salient distractors that are otherwise irrelevant for the task at hand can disrupt top-down control and capture attention (“attentional capture”: Theeuwes, 1992; Yantis & Jonides, 1990) as well as our eyes (“oculomotor capture”: Theeuwes, Kramer, Hahn & Irwin, 1998; Theeuwes, Kramer, Hahn, Irwin, & Zelinsky, 1999; Yantis & Egeth, 1999). Such findings demonstrate that while we are often in control of how and what we search, bottom-up processes can affect our performance not only independently of top-down processes but even in spite of them (but see Bacon & Egeth, 1994; Folk, Remington, & Johnson, 1992; Leber & Egeth, 2006, for the extant discussion on this matter).

Influential theoretical as well as computational models on attentional control assume that all signals driving attention converge on a priority map in the brain (Bisley, 2011; Itti & Koch, 2001; Theeuwes, 2010; Wolfe, 2007; Zelinsky & Bisley, 2015). This priority map represents an integration of both top-down and bottom-up signals in a common priority space. The highest peak of activation on the priority map corresponds to the spatial location that is most likely attended next. According to these models, attention is predominantly spatial (but see feature-based attention accounts, e.g., Maunsell & Treue, 2006; Treue and Trujillo, 1999) and hierarchical in the sense that the specific location with the highest activity on the priority map is most likely to attract attention first in a winner-takes-all fashion. Since physical stimulation may vary and top-down and bottom-up processes unfold differently over time, the priority map is subject to a constantly ongoing change in which peak activity on the map is shifting from one location to the next.

At the heart of the priority map concept lies the idea that its activity profile determines the outcome of the brain’s intrinsically emergent property of competition for neural representation (Desimone, 1998; Desimone & Duncan, 1995). Selectivity of attention, according to the theory of biased competition, resolves the competition by biasing it in favor of the stimulus with the highest priority signal. The notion that competition for neural representation is a dominant attribute along the entire hierarchy of the visual cortex might also explain why many different areas have been suggested to constitute a priority map (e.g., Balan & Gottlieb, 2006; Bisley & Goldberg, 2010; Li, 2002; Mazer & Gallant, 2003). Although early models aimed to explain the attentional modulations in the neuronal response, the idea of competition for selection is now also reflected in many behavioral models. For instance, the biased competition model of oculomotor control by Godijn and Theeuwes (2002) assumes that eye-movement control is driven by activity levels within the hierarchical priority map, or saccade map, presumably located in the superior colliculus (SC). Activity on the saccade map is assumed to spread to neighboring locations but inhibits activity of more distant locations through lateral inhibition. When the activity somewhere on the map passes a certain threshold, a saccade toward that location is executed. Modulations of activity are thought to occur through an integration of early bottom-up and late top-down signals that evolve after the onset of the search display (e.g., Mulckhuyse, Van der Stigchel, & Theeuwes, 2009; Trappenberg, Dorris, Munoz, & Klein, 2001; van Zoest, Donk, & Theeuwes, 2004). Accordingly, early in time, a salient distractor will generate activity within the saccade map corresponding to a particular location, which in turn will inhibit activity at more distance locations. Only later in time, top-down processes may permit the suppression of this distractor activation, allowing activity to build up at the location of the target. In line with this model, erroneous saccades to physically salient distractors are particularly observed in quickly initiated eye movements.

In short, top-down attentional control enables us to focus on objects and events that are relevant to our immediate goals. Volitional shifts of spatial attention involve disengaging of attention from the current focus, orienting attention to a new location in space, and selectively processing information at that location. The key aspect of this type of control is that it is volitional in origin; disengaging and engaging of attention is intentionally controlled by the observer and thus driven by the task set, intentions and/or other top-down goals. If attention is captured by a salient event even when we had no intention to direct attention toward it, one speaks of exogenous or bottom-up orienting (see Theeuwes, 2010, 2013). The activations associated with these types of attentional control are integrated within a common priority map, whose activity profile changes over time from early bottom-up to later top-down control.

Even though this dichotomy between top-down and bottom-up control can account for a wide range of observations, Awh et al. (2012) recently pointed out that there are many instances in which it falls shorts in explaining behavior. In these instances, it seems that selection is neither controlled in a top-down (volitional) way nor in a bottom-up (salience-driven) way. Instead, the history of attentional deployments can elicit selection biases prioritizing stimuli that are neither salient nor relevant for the task at hand. In the following section, we discuss evidence for one class of this history of attentional deployments which is actively shaped by rewarding experiences that went or still go along with attentional selection. First, we will review literature that linked top-down attentional control to reward incentives, before we move on to discuss the recent surge in studies that plead for an influence of reward history that goes beyond and above the influence of top-down and bottom-up control.

## Attention and reward

As early as 1911, Thorndike postulated the law of effect (Thorndike, 1911, p. 244), which stated that behavioral responses soliciting a reward will become strengthened or habitual (cf. instrumental or operant conditioning). Not long after Thorndike, Pavlov (1927) observed that stimuli reliably predicting reward would eventually start to elicit a behavioral orienting response. While overt behavior and the processes of learning were initially the focus of research, later, other researchers were trying to understand what internal states accompanied the strengthening of response patterns due to reward (e.g., Simon, 1967). This led to theories on how rewards motivate behavior as well as internal states (e.g., Berridge & Robinson, 1998; Mackintosh, 1975; Pearce & Hall, 1980). What has become evident in recent studies on attention is that similar principles as those formulated in classic theories may hold for how reward influences attention.

### Reward as a motivational incentive

In the animal world, survival depends on how effective animals are in obtaining rewards such as water, food or sex. Typically, when reward is expected (for instance, as an incentive for performing a specific task), animals (including humans) perform better than when no reward is expected (e.g., Wise, 2004). Beneficial influences of incentive reward are observed in a variety of tasks and impact behavior in different ways, irrespective of whether it is considered a primary (e.g., food or water) or secondary (e.g., money or social approval) reinforcer.

Traditionally, incentive rewards have been studied in the context of cognitive control, which refers to the set of functions that encode and maintain the representation of the current task set (i.e., currently relevant stimulus–response and action–outcome associations or goals; Botvinick & Braver, 2015). This set of functions is comprised of a variety of components involving memory, action, and attention. The influence of incentive reward on any of these components has been a topic of extensive research, which revealed a strong connection between reward and cognitive control. For instance, incentives have been shown to improve response preparation and inhibition (e.g., Boehler, Schevernels, Hopf, Stoppel, & Krebs, 2014; Mir et al., 2011), enhance the control of episodic memory encoding and retrieval (e.g., Patai, Doallo, & Nobre, 2012) and increase adaptation to errors or other cognitive conflict (e.g., Braem, Verguts, Roggeman, & Notebaert, 2012; >Stürmer, Roland Nigbur, & Sommer, 2011; for a more detailed review, see Botvinick & Braver, 2015). More important for the scope of this review is, however, that incentive rewards have also been demonstrated to strongly influence attentional control. For instance, Engelmann and Pessoa (2007) showed in a spatial cueing task (Posner, Snyder, & Davidson, 1980), that participants had a higher detection sensitivity in blocks in which they were informed that good performance was rewarded compared to blocks in which they knew it was not (see also Engelmann, Damaraju, Padmala, & Pessoa, 2009; Milstein & Dorris, 2007; Small et al., 2005). Padmala and Pessoa (2010) demonstrated in a Stroop task that, when reward was at stake, target selection was facilitated on distractor-congruent trials and interference in target selection was smaller on distractor-incongruent trials. Studies like these suggest that incentives improve performance via motivating strategic processes such as the preparation of an appropriate attentional or oculomotor orienting response (e.g., Bucker & Theeuwes, 2014; Engelmann & Pessoa, 2007; Milstein & Dorris, 2007; Sawaki, Luck, & Raymond, 2015; Small et al., 2005) or facilitating selective attenuation of irrelevant distractors (i.e., attentional filtering; Padmala & Pessoa, 2008, 2010).

Neuroimaging studies have linked incentive-induced changes in neuronal activity to brain regions and electrophysiological markers that are usually observed when manipulating traditional factors of attentional control. For example, an fMRI study by Small et al. (2005), which used a spatial cueing task, observed widespread activations of task-specific regions in response to reward-signaling cues presented prior to a given trial. As would be expected from a cueing task, many brain areas considered to be important in the orienting of visuospatial attention showed increased activity when reward was at stake. The implication, then, is that strategic and task-specific processes (i.e., the orienting of spatial attention) were more vigorously prepared throughout the interval between cue and search display onset and, as a consequence, task performance improved. Corroborating electrophysiological evidence comes from a study by Sawaki and colleagues (Sawaki, Luck, & Raymond, 2015), who observed reduced alpha power for high-reward relative to low-reward trials during a preparatory interval (between reward cue offset and search display onset). According to the authors, this reflected visual alertness, or increased cognitive effort to focus attention, for the upcoming search task. Indeed, subsequent to the search display onset, the target-elicited N2pc component, thought to reflect attentional selection and originating in extrastriate visual areas such as V4 (e.g., Eimer, 1996), was smaller on high-incentive than on low-incentive trials. This is consistent with the typical finding of a reduced N2pc in easy versus hard search tasks (Eimer, 1996; Luck & Hillyard, 1994). Sawaki and colleagues argued that the preparation interval allowed for more efficient attentional processing when more reward was at stake, which reduced the need for focused attention.

In contrast to the previous studies, in which attentional control was more broadly affected, another type of studies showed a selective influence of incentive reward, which is specific to the perceptual features or the spatial location of targets associated with reward. In such studies, participants typically receive reward if they successfully attend a specific stimulus or its location, thereby forming an association between a stimulus (feature) and reward (Kiss, Driver, & Eimer, 2009; Krebs, Boehler, & Woldorff, 2010; Kristjánsson, Sigurjónsdóttir, & Driver, 2010; Serences, 2008). Here, too, performance improves when either a feature of a valid cue or the target itself is associated with a larger reward. For instance, in Kiss et al. (2009), participants were rewarded for searching for a color singleton while the magnitude of reward depended on the color of that singleton. Clearly distinct from other incentive reward studies was that not only the appropriate response but also the (magnitude of the) reward itself was uncertain up until the presentation of the target since trials with different outcomes were interleaved rather than blocked. Nevertheless, the results showed faster identification for high-reward than for low-reward targets. Importantly, electrophysiological recordings showed that the N2pc component was also modulated by reward magnitude. The N2pc was earlier and larger in response to high-reward targets compared to low-reward targets. At first glance, these findings seem to be at odds with Sawaki et al. (2015), who found a smaller N2pc in high reward trials. However, the rewarded stimulus in their study appeared well before the actual search display, which allowed for optimal preparation. Since optimizing preparation for specifically high-reward trials was not possible and the search display was more cluttered in Kiss et al., search difficulty may have increased such that there was a stronger need for selective processing. Nonetheless, findings from studies in which trial-by-trial reward is uncertain until the onset of the target not only support the notion of preferential processing but also show a prioritized attentional state for reward-signaling targets when selection among multiple competing stimuli is necessary.

Irrespective of whether reward was signaled prior to a block of trials, prior to an individual trial, or contingent on the target, the beneficial influence of reward in all these studies can be readily explained in terms of ongoing top-down attentional control processes (Maunsell, 2004; Stănişor, van der Togt, Pennartz, & Roelfsema, 2013). In some studies, time intervals allowed for alertness or strategic preparation of efficient attentional control. In other studies, participants strategically searched for the stimulus signaling reward because it was either the target or in any other way relevant for task performance (e.g., because it was bound to the spatial location of the target). Additionally, the reward-associated stimulus was also the motivated target as it is the intrinsically motivated—in some studies even instructed—goal to obtain as much reward as possible. Conjointly these studies therefore provide evidence for flexible incentive-induced modulation of the top-down task set. The prospect of reward can either broadly strengthen the task set when reward is generally at stake, or more selectively affect components of the task set, such as the representation of the target template or appropriate response, when reward is associated with a specific feature or the location of the target. Concurring evidence for this notion comes from recent neuroimaging studies that demonstrated more accurate decoding of task-set representations in frontoparietal regions on reward relative to no reward trials (Etzel, Cole, Zacks, Kay, & Braver, 2015; Wisniewski, Reverberi, Momennejad, Kahnt, & Haynes, 2015). Critically, it was shown that better task decoding, as demonstrated by higher multivoxel pattern analysis classification accuracy, mediated changes in behavioral performance due to incentive reward. More specifically, stronger task-set representations (measured as higher classification accuracy) in frontoparietal regions due to incentive reward predicted higher accuracy on the task (Etzel et al., 2015).

In short, studies have provided evidence for a widespread influence of incentive reward on executive functioning and, more broadly, cognitive control which is also observed on the level of attentional control (Botvinick & Braver, 2015). The prospect of earning reward may lead to strategic changes in top-down attentional control which subsequently have a beneficial effect on perceptual as well as attentional processing. This notion is in line with findings showing that incentives ultimately benefit attentional processing (Kiss et al., 2009; Sawaki et al., 2015) as well as enhance the perceptual representation of reward-associated targets (Seitz, Kim & Watanabe, 2009; Serences, 2008). Recent evidence shows that these changes in processing may be mediated by an enhancement of endogenous task-set representations in frontoparietal regions (Etzel et al., 2015; Wisniewski et al., 2015), which promotes task performance either more broadly or selectively, depending on the specific task demands.

### Reward history can counteract top-down processes

In the studies discussed in the previous section, rewards were always congruent (i.e., not detrimental) to task demands. Observing performance benefits when either reward was generally at stake (i.e., in a reward block) or directly tied to the target or otherwise task-relevant stimulus might therefore not come as a surprise: It makes sense to strategically prioritize the reward-associated stimulus for attentional selection in order to ensure a higher rate of success with the added benefit of earning larger reward. However, many studies cannot convincingly exclude the possibility that the observed reward effect reflects, at least in part, a more automatic influence of the recent history of reward-driven selection. Reward-based selection history may in fact have affected ongoing selection either along or even without top-down control. In other words, the finding that reward manipulations were typically congruent with task demands or the task set does not necessarily mean that top-down control was the source of the observed effect.

In this section, we discuss recent studies in which reward is pitted against known top-down or bottom-up processes. These studies shed light on the question of whether a reward signal competes with other attentional control processes for attentional selection. We will start by reviewing evidence from three different type of studies designed to disentangle reward from top-down and bottom-up control before we briefly discuss the role of dopamine in learning reward associations. The evidence from these studies suggests that reward can shape covert as well as overt attention through selection history.

#### Short-term or intertrial priming by reward

An association between a stimulus and a reward implies the involvement of some sort of a memory process and the notion that memory processes are capable of shaping attentional selection is not new. For instance, (intertrial) priming is a powerful way by which current selection can be biased toward stimuli that have been selected in the very recent past (e.g., on the previous trial) irrespective of other concurrently active attentional control processes (Maljkovic & Nakayama, 1994; Tulving & Schacter, 1990). Only recently, however, perceptual priming has been shown to be further modulated by reward outcomes.

Early evidence of reward’s influence on priming was provided by experiments of Della Libera and Chelazzi (2006). They demonstrated modulations of global and local stimulus priming by reward, suggesting that stimuli, for whose inhibition participants were rewarded, were subsequently harder to select. Even more convincing evidence was provided by Hickey and colleagues (Hickey, Chelazzi, and Theeuwes, 2010a). This study was based on the additional singleton paradigm of Theeuwes (1991b, 1992): a speeded task in which participants have to search for a shape singleton object. The critical distractor, or additional singleton, is defined by its physically salient color (e.g., a red among green shapes) and is task irrelevant on every trial. The traditional finding of this task is that the additional singleton distractor captures attention in a bottom-up fashion (Theeuwes, 1991b, 1992). Since target-associated and distractor-associated features can swap between trials, another classic finding is intertrial priming. If target-associated and distractor-associated features swap from one trial to another, capture by the distractor is larger since its feature was recently associated with the target (Pinto, Olivers, & Theeuwes, 2005). Crucially, however, using this paradigm Hickey and colleagues showed that reward mediates intertrial priming. When participants received a high reward, they were quicker in searching the target on the subsequent trial if no swap between the color associated with the target and the distractor occurred, but slower if a swap occurred. Conversely, when participants received a low reward, they were slower in searching the target on the subsequent trial if no swap occurred, but quicker if a swap occurred. Hickey and colleagues explained this finding in terms of relative revaluation and devaluation of perceptual features characterizing the target due to short-term reward history.

Remarkable about intertrial reward priming is its seemingly automatic nature. This notion was particularly evident in a follow-up experiment (Hickey et al., 2010a) in which participants engaged in the same task but, this time, were explicitly told that whenever a high reward was earned, the colors associated with the target and the distractor would swap. There was, therefore, absolutely no incentive to strategically search for the feature recently associated with reward as it would provide only redundant (i.e., already known) information. As a matter of fact, attending it would hamper target search and reward payout because it would be more difficult to identify the target in time. In spite of that, reward-mediated intertrial priming was observed: participants could not help but preferentially attend to the object of the same color as the high reward target on the previous trial even if it was no longer the target.

Strong neuronal evidence that recent reward history changes the perceptual and attentional processing of the reward-associated feature was provided from electrophysiological recordings in the same study (Hickey et al., 2010a). Not only was there an enhanced P1 component, which is thought to reflect increased early visual processing in extrastriate cortex without attentional modulation (cf. Hillyard, Vogel, & Luck, 1998; Luck & Hillyard, 1994), but also a larger N2pc in response to the stimulus recently associated with high reward. This suggests that, by mediating perceptual priming, reward affects the representation of a stimulus rendering it more salient within the visual system.

Intertrial reward priming has since then been demonstrated to affect covert search even under more realistic circumstances such as when searching for an object (e.g., a car) in a natural scene (e.g., a busy city street; Hickey, Kaiser, & Peelen, 2015). Overt search has also been shown to be affected by intertrial reward priming as evidenced by modulations in oculomotor capture as well as saccadic trajectories due to reward (Hickey & van Zoest, 2012, 2013). These reports underscore the large degree of generalizability of intertrial reward priming and its immediate effect on visual selection.

In summary, studies on intertrial reward priming provide evidence that reward mediates perceptual priming independently of active top-down processes. They furthermore support the idea that reward-based selection history impacts stimulus representation: reward changes the stimulus’ pertinence to the visual system, prioritizing its perceptual and attentional processing irrespective of whether this serves top-down control or the acquisition of reward.

#### Persistent reward biases and value-driven attentional capture

Intertrial reward priming studies provide some evidence that rewarding attentional selection of a specific stimulus (feature) creates reward-based selection history, which influences the next selection episode. Albeit short lasting (i.e., selection benefits at the next trial), this cannot be readily understood in terms of online active top-down processes. However, one may argue that because participants received reward throughout the entire experiment and were every so often rewarded for the selection of the feature that would shortly thereafter function as distractor, intertrial reward priming is merely a demonstration of the limitations in flexibly adjusting top-down control. Moreover, the stimuli imbued with short-term reward-based selection history were often physically salient, mandating attentional selection independent of reward-based selection history.

Other lines of research aimed to address these and other drawbacks of investigating the influence of reward on attentional selection in the context of intertrial priming. For instance, some early studies focused on investigating a long-lasting influence of reward-based selection history and whether it could be shaped by rewarding only certain responses. In one of such studies, Della Libera and Chelazzi (2009) split up the experiment into two sessions, separated by 5 days. During the first, or “training,” session participants were rewarded for performing a shape-matching task. Although participants were told that trial-by-trial reward payout for correct judgements was based on their performance, it was in fact (and unbeknownst to the participants) biased toward specific relationships of the shape and its role in the display (i.e., either it was a target or a distractor). Certain shapes were associated with high reward when appearing as targets, while others were associated with low reward when appearing as targets. Yet others were associated with high reward when appearing as distractors, while others were associated with low reward when appearing as distractors. When any of these shapes appeared in a different role, its reward payout was not biased toward a specific magnitude but instead randomly but equally often high or low. In the subsequent “test” session, participants engaged in the same task as during training but, critically, could no longer earn any reward. The results showed that performance during the test session was modulated as a function of the specific role of the shapes during the training session. Distractor shapes that were associated with high reward when they were targets during training slowed correct responses more so than distractor shapes that were associated with low reward when they were targets during training. Conversely, distractor shapes that were associated with high reward when they were distractors during training sped up correct response compared to distractors that were associated with low reward when they were distractors during training. Interestingly, a different effect was observed in a follow-up study in which participants were told that reward payout was completely random (Della Libera, Perlato, & Chelazzi, 2011). Under these circumstances, distractor shapes previously associated with high reward slowed responses during the test session, regardless of their specific role during training.

In short, the experiments by Della Libera and colleagues show that stimuli for whose selection one has received larger reward persist to affect selection by forming potent distractors even if they are no longer associated with reward. In contrast, stimuli for which one has received larger reward when successfully ignoring them continue to be more easily ignored even if reward can no longer to be earned. This shows that reward-based selection history affects selection for considerably longer than the immediately following trial (cf. intertrial priming). More importantly, it suggests that the reward priority signal can be shaped under certain conditions such that its influence on selection is enhanced or suppressed. Even though these findings are important, specific aspects remained unaddressed. For instance, one may argue that any influence of reward-based selection history was not sufficiently decoupled from task-relevance in these studies. Indeed, all stimuli were equally often presented as distractors and targets not only during training but also in the test session. Similarly, the nature of the selection bias remained unclear (i.e., whether the reward-associated stimuli were more likely to capture attention or to delay the disengagement of attention). Finally, the observation that distractors imbued with reward-based selection history did only compete with target selection when the training and test session were identical (and not when they were different) questions how generalizable these findings are (cf. Experiment 1 and 2 in Della Libera & Chelazzi, 2009).

Support for the notion of a persistent influence of reward-based selection history was provided by Anderson and colleagues (Anderson, Laurent, & Yantis, 2011). Their experiments were also split up into a training and a test session. During training, participants searched for a red or a green circle presented among differently colored nontarget circles. One target color was more often indicative of high reward while the other was more often associated with low reward. In the subsequent test session, participants searched for a shape singleton presented among randomly colored nontarget shapes (cf. additional singleton task; Theeuwes, 1992). Critically, on half of the trials, one of the nontargets was rendered in either one of the colors of the target during the training session. During the test session, Anderson et al. observed an increase in the time to find the target when a nontarget had a color that was previously associated with a high reward relative to when none of the previously reward-associated colors was present. Importantly, this effect was observed even though participants were explicitly instructed to ignore any color information and informed that they could no longer receive reward during the test session. Search time moreover increased although the stimuli previously associated with reward were physically not more salient than any of the other nontargets. In fact, the target was always the most physically salient item in the display. The authors argued that the increase in search time was due to attentional capture by the stimulus previously associated with high reward, which could neither be explained in terms of top-down nor in terms of bottom-up processes. Although initially only demonstrated when both sessions occurred on the same day, subsequent studies showed that such interference can be observed considerably longer—even up to 6 months after the training session (e.g., Anderson & Yantis, 2013).

Failing and Theeuwes (2014) expanded the evidence for involuntary attentional orienting toward previously reward-associated stimuli by demonstrating performance costs and benefits in the context of a spatial cueing experiment. Finding such costs and benefits is considered direct evidence for shifts of spatial attention (Posner & Cohen, 1984; Posner et al., 1980) precluding alternative explanations of interference in search performance due to, for instance, nonspatial filtering costs (cf. Kahneman, Treisman, & Burkell, 1983). Their findings moreover demonstrated that capture is truly the result of reward-based selection history rather than selection history per se. This was an important refinement of Anderson and colleagues’ findings (also Rutherford, O’Brien, & Raymond, 2010) since they initially only reported a significant difference between trials with distinct selection history but not between trials with identical selection history (although a recently published reanalysis of the data and direct replication of the study did find a difference between high-reward and low-reward trials; see Anderson & Halpern, 2017). Failing and Theeuwes found that previously rewarded stimuli indeed captured attention in spite of concurrently presented stimuli that were equally often selected but not rewarded during the training session. Consistent with the assumption that it was reward learning during training that caused capture during the test session, participants who showed a larger reward effect during learning (i.e., facilitated selection of the reward-associated target) also showed a larger reward effect during the test session (i.e., more capture by the reward-associated cue).

Reward-based selection history created during a separate training session has also been demonstrated to influence overt search. Target objects associated with relatively high reward during a training session invoke more oculomotor capture compared to objects previously associated with low reward when presented as a task-irrelevant distractor in a subsequent test session. Oculomotor capture due to reward-based selection history has been observed when participants are unconstrained where to look at (Anderson & Yantis, 2012; Bucker, Silvis, Donk, & Theeuwes, 2015) or when instructed to search for a target defined by a different feature than the one previously associated with reward (Theeuwes & Belopolsky, 2012).

Yet another line of research has demonstrated that temporal attentional selection is influenced by reward-based selection history in a very similar way. Raymond and O’Brien (2009) were first to demonstrate that reward affects temporal selection in the attentional blink paradigm (AB; Raymond, Shapiro, & Arnell, 1992). In the AB task, participants are usually presented with a stream of stimuli in which each stimulus is presented for only a short duration at the center of fixation. Participants typically have to detect two targets or discriminate between the features of two targets embedded in the stream. Performance on the second target is typically severely deteriorated when it is presented shortly (around 200 ms) after the first target, compared to when the interval between the presentation of the targets is longer (e.g., 800 ms; Dux & Marois, 2009; Shapiro, Raymond, & Arnell, 1997). As the moment at which the target is presented is uncertain, these tasks clearly demonstrate the limits of available attention across time.

In Raymond and O’Brien’s (2009) experiments, participants first learned associations between different face stimuli and reward magnitudes (wins, neutrals, and losses) in the context of a choice game. Then, in a subsequent test session in which there was no longer any reward, participants saw two targets (T1 and T2) temporally separated by either a short or a longer interval. When participants were shown a face from the training session as T2, its successful recognition depended on the expected value coupled to it during training with an expected reward or loss improving recognition above recognition performance for faces tied to no expected value. Critically, when both targets were separated by a short interval, only the face pictures previously coupled to reward remained unaffected by the interval between target presentations. When the order of targets was swapped in the test session of a follow-up experiment such that the reward-associated faces were presented as T1, T1 performance was still modulated by reward but performance on T2 (an unseen nonface stimulus) was not. Consequently, Raymond and O’Brien concluded that recognition is better for stimuli previously coupled to a high probability outcome of earning or losing but only reward-associated stimuli are able to overcome the AB (see also Yokoyama, Padmala, & Pessoa, 2015).

Finding that recognition of T2 was unaffected by the reward associated with T1 is somewhat surprising in light of the substantial evidence for a prioritized attentional status of reward-associated stimuli at the expense of others (including physically more salient targets). The reward-dependent prioritized status of a stimulus should come at a larger cost for subsequent processing of other task-relevant stimuli, particularly since the AB reflects failed attentional suppression during T1 processing (Martens & Wyble, 2010; Taatgen, Juvina, Schipper, Borst, & Martens, 2009; Wyble, Bowman, & Nieuwenstein, 2009). In other words, one would have expected that T2 performance deteriorates as a function of the reward association of T1 assuming that reward affects availability of attention across time in a similar way as it affects spatial attention. Failing and Theeuwes (2015) speculated that Raymond and O’Brien found no interference in T2 performance because attentional processing of T2 was already at maximum priority since attending these stimuli was task-relevant throughout the entire experiment.

To address this issue, Failing and Theeuwes (2015) conducted a series of experiments in which the reward-associated stimulus was always a task-irrelevant distractor during the test session. In their experiments, participants had to detect the presence of a single target (a picture of a designated category of scenes, e.g., forest scenery) embedded in a stream of different stimuli (pictures of another category of scenes, e.g., field scenery) throughout the test session. In two thirds of the trials, a previously rewarded stimulus (i.e., a picture of a third or fourth category of scenes, e.g., water and mountain scenery) was embedded as a distractor in the stream. The results showed interference in the sensitivity to detect the target when a previously reward-associated stimulus was present even though these stimuli were completely task-irrelevant and no longer signaled any reward. Importantly, the degree of interference depended on the magnitude of reward previously associated with the distractor—with high reward causing the strongest interference. Particularly remarkable was that this effect was found even though reward was associated with complex visual scenes of a given semantic category rather than simple features and that it generalized to unseen stimuli from the same semantic category (see Experiment 2, Failing & Theeuwes, 2015). Given the severely limited presentation duration of each stimulus, this suggests that reward either affects highly complex sets of features or may act on the level of gist perception. With respect to the broader picture, these studies clearly demonstrate that reward-based selection history not only affects spatial but also temporal attention.

Although little is known about the neuronal underpinnings of how stimuli imbued with reward-based selection history affect temporal attention, electrophysiological studies using training-test session designs to investigate spatial attention demonstrate that such stimuli enjoy a preferential status in perceptual and attentional processing when reward can no longer be earned. For instance, Qi and colleagues (Qi, Zeng, Ding & Li, 2013) showed that the N2pc component was larger in response to distractors previously associated with a high reward. Intriguingly, they observed no modulation of the N2pc on fast trials but instead a larger Pd component, which is thought to index attentional suppression (e.g., Sawaki, Geng, & Luck, 2012). The authors concluded that only if successfully suppressed, selection of the stimulus previously associated with high reward could be prevented. Converging evidence from another study that demonstrated modulations in the P1 component due to reward-based selection history up to 7 days after the initial training session (MacLean & Giesbrecht, 2015). In line with the idea of increased perceptual processing, previously reward-associated stimuli elicited a larger P1.

Neuroimaging studies using separate training and test sessions provide evidence that changes in perceptual and attentional processing of previously reward-associated stimuli can be localized in areas critically involved in representing selection priority. For example, Anderson and colleagues (Anderson, 2016b; Anderson, Laurent, & Yantis, 2014) showed that previously reward-associated stimuli evoke activity in extrastriate cortex as well as the intraparietal sulcus (IPS), both critical to attentional control (Serences et al., 2005). Since the IPS has been suggested to map priority signals for attentional selection, this indicates that the reward-based selection history signal persists to be neuronally represented even if reward is no longer available.

The majority of behavioral and neuroimaging studies make a strong case for what has since been referred to as value-driven attentional capture (VDAC). A great wealth of studies has continued to explore the specificity and extent of this long-lasting reward effect demonstrating its influence on a variety of low-level features as well as on more complex stimuli such as faces, objects, or visual scenes (for excellent reviews, see Anderson, 2013, 2016a; Chelazzi, Perlato, Santandrea, & Della Libera, 2013; Le Pelley, Mitchell, Beesley, George, & Wills, 2016; Vuilleumier, 2015). Growing evidence demonstrates that prioritization due to reward-based selection history may not be limited to features but is also observed for spatial locations (Chelazzi et al., 2014; Hickey, Chelazzi, & Theeuwes, 2014; but see Leber & Won, 2016; Won & Leber, 2016), modulates contextual cueing (Pollmann, Eštočinová, Sommer, Chelazzi, & Zinke, 2016; Tseng & Lleras, 2013), affects processes related to the processing of time (Failing & Theeuwes, 2016; Hickey & Los, 2015; Rajsic, Perera, & Pratt, 2016) as well as sound (Anderson, 2016c; Asutay & Västfjäll, 2016) and develops differently in distinct subpopulations (Anderson, Faulkner, Rilee, Yantis, & Marvel, 2013; Anderson, Kronemer, Rilee, Sacktor, & Marvel, 2016; Anderson, Leal, Hall, Yassa, & Yantis, 2014; Roper, Vecera, & Vaidya, 2014).

An important difference of all the above discussed studies to those concerned with intertrial reward priming is that the stimuli no longer signaled any reward but attentional selection was still—even after months—affected by the reward associations. In VDAC studies in particular, the stimuli that had previously been associated with a high reward were task-irrelevant and physically nonsalient throughout the designated test session but still enjoyed a prioritized status in selection. The fact that there was, according to classic theories on attentional control, no reason to attend these stimuli, neither in the sense of top-down or bottom-up attentional control nor in terms of maximizing reward outcomes, provides strong evidence for a separate class of priority signal in attentional selection. This priority signal is shaped by reward-based selection history and has a long-term impact on the perceptual as well as attentional processing.

#### Attentional capture via Pavlovian reward learning

It seems that one key factor in the experimental design of the training-test session studies is the instrumental value of the reward-associated stimuli during the training session. In other words, similar to the design of incentive reward studies, reward is obtained when successfully selecting the reward-associated target throughout the training session. Prioritized selection of the previously reward-associated stimulus as observed during the test session can therefore be understood in terms of an instrumentally conditioned response (cf. Thorndike, 1911) or habitual orienting response instigated by reward (“attentional habit”; see Anderson, 2016a; Le Pelley et al., 2016) that is carried over from the training session. This impact of instrumental conditioning on attentional selection is particularly highlighted by the experiments of Della Libera and colleagues, in which current selection was shaped according to the specific responses that led to the highest possible reward payout.

Recent efforts focused on investigating whether the impact of reward-based selection history can only be observed as the consequence of an instrumental relationship or, alternatively, also due to a Pavlovian relationship between stimulus and reward. VDAC studies raised this question specifically, as their findings can be equally well accounted for by either of the two conditioning mechanisms. In early reports concerned with Pavlovian associations, reward signals were rendered orthogonal to top-down task relevance throughout the entire experiment. For instance, in one primate study, the reward-signaling cue that preceded the onset of the target could appear at but was never predictive of the location of the target (Peck, Jangraw, Suzuki, Efem, & Gottlieb, 2009). These cues had no instrumental value since only the successful target selection garnered the signaled reward. Nonetheless, Peck et al. observed that such reward-signaling cues biased oculomotor selection in monkeys. In line with the idea that an association with reward changes the stimulus representation in the visual system, neurons in the lateral intraparietal cortex (LIP; i.e., the monkey analog of the human IPS) exhibited preferential firing for cues that consistently predicted reward. However, while the reward signal may have been orthogonal to the location of the target, strategically prioritizing selection of reward-associated stimuli was also not tied to any costs for task performance or reward payout; especially because the reward-signaling cue preceded the target onset and thus did not immediately compete with the target. Other research suggests that primates strategically search for the reward-associated cue under such circumstances (Bromberg-Martin & Hikosaka, 2009).

A recent series of studies by Le Pelley and colleagues investigated whether a reward-signaling stimulus competes for attentional selection even if it never coincided with the target location on a given trial and even if its selection never resulted in reward (Le Pelley, Pearson, Griffiths, & Beesley, 2015; Pearson, Donkin, Tran, Most, & Le Pelley, 2015; for a conceptually similar approach in the context of a training-test session paradigm, see Mine & Saiki, 2015, Experiment 2). Therefore, there was not only never a benefit from selecting the reward-signaling stimulus under these circumstances but actual costs equivalent to deteriorated task performance and less reward payout. The experimental paradigm used in these studies was based on the additional singleton task (Theeuwes, 1992): participants searched for a shape singleton while a color singleton, varying in color and present on two thirds of the trials, had to be ignored. The critical manipulation was that the color of the singleton distractor signaled the magnitude of the reward that could be earned if participants would give the correct response within a given time limit. As there was no separate training session, the stimulus signaling reward was task-irrelevant throughout the entire experiment and was also never required to be selected in order to obtain the reward. Le Pelley and colleagues reasoned that if any capture by that stimulus was to be found, it must have been due to a Pavlovian association between the distractor and the reward it signaled (Le Pelley et al., 2015; Pearson et al., 2015). Indeed, the results showed that attentional capture, measured as the interference in RT when comparing distractor versus no distractor trials, was larger when the distractor signaled a high compared to a low reward. In a follow-up eye movement study (Experiment 3 in Le Pelley et al., 2015) in which reward was omitted if participants would look at the reward-signaling color singleton, similar results were obtained. Here, too, a color singleton object which was task-irrelevant throughout the entire experiment caused more often an omission of reward when it signaled high relative to low reward.

Even though these results are compelling, the fact that the reward-signaling distractor was always physically salient makes it difficult to conclude that involuntary capture was entirely due to Pavlovian reward associations. Indeed, this raises the possibility that if the reward-signaling stimulus is not physically salient, one does not observe any difference in attentional capture by reward-signaling stimuli. Failing and colleagues (Failing, Nissens, Pearson, Le Pelley, & Theeuwes, 2015; Failing & Theeuwes, 2017; see also Bucker, Belopolsky, & Theeuwes, 2015) addressed this issue by demonstrating that even if task-irrelevant but reward-signaling stimuli are physically nonsalient throughout the entire experiment, one does observe attentional (Failing & Theeuwes, 2017) as well as oculomotor capture (Failing et al., 2015). Critically, and further supporting the notion of an involuntary bias toward reward-signaling stimuli, particularly the early first saccades were shown to be prone to capture by high-reward relative to low-reward distractors (Failing et al., 2015). It is worth mentioning that participants in these studies were typically informed about the association between the task-irrelevant and physically nonsalient stimulus and the reward it signals. Although informing participants about the stimulus–reward association seems to influence the likelihood of whether task-irrelevant and physically nonsalient stimuli that signal reward summon attention, it does not seem to be mandatory (Bucker & Theeuwes, 2014; see Experiment 6 in Failing & Theeuwes, 2017).

A recent study investigated the role of reward in temporal attention when a particular stimulus merely signaled the availability of reward and thus never had instrumental value (Le Pelley, Seabrooke, Kennedy, Pearson, & Most, 2017). In this study, participants had to identify the orientation of a rotated picture in an RSVP stream of upright pictures presented at fixation. The amount of reward that could be earned or lost on an individual trial was signaled by an upright distractor picture that was presented either a short or a long interval before the target. The results showed that participants’ accuracy dropped significantly when the distractor signaled that reward could be earned compared to when no reward was signaled or when such a distractor was absent. This was not only the case when participants were explicitly informed about the reward-signaling property of the distractor but also when no such information was given. It is important to note that the reward-associated distractor caused interference even though it was not necessary to select that stimulus to earn the reward but, instead, selecting it decreased the likelihood of reward payout. Also, interference remained when participants were explicitly informed that distractor selection would result in reduced reward. This shows that even if reward-signaling stimuli are task-irrelevant throughout the entire experiment and attending them is detrimental to the goal of obtaining reward, they interfere with temporal attention.

In short, the previously discussed studies demonstrate that reward can modulate capture of physically salient but otherwise task-irrelevant stimuli even if the selection of such stimuli never results in but actually hinders reward payout. Finding modulations of salience-induced capture due to reward history has been referred to as value-modulated attentional capture (Le Pelley et al., 2015; Pearson et al., 2015). Later reports provided evidence for capture without the need of physical salience to drive initial selection. Indeed, capture also occurs for physically nonsalient and task-irrelevant stimuli when their reward-association is either made explicit or implicitly learned (Bucker & Theeuwes, 2017a; Failing et al., 2015; Failing & Theeuwes, 2017; Le Pelley et al., 2017). These studies therefore provide strong evidence that not only instrumental but also Pavlovian associations can govern attentional selection priority due to reward.

### On the role of dopamine in learned reward priority

An important question regarding any influence of reward is what mediates the learning of reward contingencies. Major theories ascribe the dopaminergic systems in the midbrain, particularly the substantia nigra and ventral tegmental area, and their projections onto other brain areas such as the basal ganglia and the frontal part of the cerebral cortex, a pivotal role during reward learning. Although most theories support the idea that dopamine contributes to neuronal plasticity alongside other neuromodulators, its specific role in reward learning is still a matter of debate (e.g., Aarts, van Holstein, & Cools, 2011; Berridge & Robinson, 1998; Hikosaka, Kim, Yasuda, & Yamamoto, 2014; Roelfsema, van Ooyen, & Watanabe, 2010; Schultz, 1997). For instance, dopamine has been suggested to either mediate general functions of action generation, effort, movement, and general arousal; represent the hedonic value of reward; code a learning or teaching signal that forges associations or represents a simple reward-prediction error; or mediate the dynamic “wanting” attribute of incentive salience (for a detailed discussion on the putative functions of dopamine, see, e.g., Berridge, 2007).

There is evidence for the involvement of the dopaminergic system in the context of reward-driven modulations in attentional control. For example, similar to other studies investigating the influence of incentive reward on attentional control (e.g., Krebs, Boehler, Egner, & Woldorff, 2011; Krebs, Boehler, Roberts, Song, & Woldorff, 2012; Small et al., 2005), studies by Pessoa and colleagues demonstrated that incentive reward evoked activity in large distributed brain networks, particularly those associated with attention and dopamine release (Engelmann et al., 2009; Padmala & Pessoa, 2011; for a review, see Pessoa & Engelmann, 2010). Importantly, by employing network analysis, they showed that while those networks were largely segregated during control trials (i.e., they exhibited high within-network connectivity), there was less segregation (i.e., higher between-network connectivity) during reward trials (Harsay et al., 2011; Kinnison, Padmala, Choi, & Pessoa, 2012). In other words, given the direction of midbrain projections, dopaminergic activity due to incentive rewards might increase the coupling and integration between the networks allowing for optimal performance. Pessoa (2015) speculated that dopamine improves the signal-to-noise ratio of relevant neurons (e.g., Sawaguchi & Matsumura, 1985), which “sharpens” attentional control and, in turn, may enhance processing efficiency in target cortical and subcortical regions. Corroborating evidence comes from Noudoost and Moore (2011), who pharmacologically altered the dopamine receptor activity in the FEF of the prefrontal cortex and measured its effect on extrastriate area V4. D1 receptors in the FEF mediated prefrontal control by leading to enhanced and more selective neuronal responses in V4 in a manner that was comparable with known top-down attentional modulations.

As discussed, the influence of reward is not only restricted to modulations in strategic attentional control. Nevertheless, studies investigating the influence of stimulus-specific reward-associations on selection also indicate a vital role of dopamine even if the reward effects occur irrespective of competing top-down processes. Findings from those studies support the idea that dopamine is responsible for changes in the representation of a reward-associated stimulus. For instance, the behavioral impact of intertrial reward priming has been shown to positively correlate with a midline anterior ERP component, called medial frontal negativity, which has been suggested to reflect the assessment of motivational impact (Gehring & Willoughby, 2002; Hickey et al., 2010a). The extent to which Anderson and colleagues (Anderson, Kuwabara, et al., 2017) observed VDAC during the test session was positively correlated with the dopamine release in the right anterior caudate during the training. In other words, more dopamine release throughout the training predicted more distraction during the test. Similarly, Hickey and Peelen (2015) showed that midbrain sensitivity to reward-associated distractor objects predicted the strength of suppression of the distractor representation in object-selective cortex (OSC; i.e., lateral-occipital complex). Interestingly, they also observed modulations in other prefrontal regions, such as the orbitofrontal cortex and dorsolateral prefrontal cortex that receive direct dopaminergic input from the midbrain. This suggests that the propagation of the dopaminergic signal from the midbrain to other areas that are not part of the visual system might also be critical for the influence of reward on selection to occur. This notion was further supported by recent lesion studies. For example, Vaidya and Fellows (2015) found that while healthy controls and patients with a lesion in the lateral or the dorsomedial frontal lobe showed intertrial reward priming, patients with a lesion in the ventromedial frontal cortex (vmPFC) did not (but see Manohar & Husain, 2016). Such findings demonstrate that the specific role of the brain areas onto which the dopaminergic midbrain projects directly is complex and depends on a variety of factors, at least with respect to their contribution to attentional selection.

A limitation of the majority of neuroimaging studies on reward-induced dopamine release and attentional control is the difficulty to assess whether midbrain activity reflects anything other than the processing of immediate reward feedback. Indeed, participants in such studies are typically continuously rewarded. Studies measuring reward effects in the absence of active reward learning, such as those consisting of a separate training and test session, are of particular importance in this regard. Regardless of this crucial difference, these studies also underscore the importance of the dopaminergic system. In one fMRI study, neuronal activity in the striatum was observed to be elevated in response to distractors that were previously associated with high reward, relative to when previously reward-associated distractors were absent (Anderson, Leal, et al., 2014). Even stronger evidence comes from a recent positron emission tomography study which found that attentional capture by previously reward-associated distractors was positively correlated with the release of dopamine within the caudate and posterior putamen (Anderson, Kuwabara, et al., 2016). Likewise, the ability to resist attentional capture by such distractors was associated with the suppression of dopamine release. It is critical to understand that participants in these studies could no longer earn any reward but the previously reward-associated yet physically nonsalient and task-irrelevant stimuli still elicited a response in the dopaminergic midbrain.

Research on the role of dopamine in reward-based selection history is still in its infancy, but the above findings blend well with all types of reward studies and major theories on reward learning and the role of dopamine. However, they also illustrate that answering the question on the functional role of dopamine is not simple as they still support multiple interpretations. Because dopamine has been shown to be modulated for stimuli previously associated with reward even when reward is no longer given (i.e., during a test session; see Anderson, Kuwabara, et al., 2016), it seems nonetheless unlikely that it merely reflects the hedonic value of reward. For similar reasons, it seems unlikely that the dopaminergic response during the reward-free test session reflects general arousal as one would expect arousal to be differently affected only when actual reward is expected. Moreover, general arousal is a more sluggish response that is unlikely to change within the short time window of reward cue presentations usually seen in visual search task (shorter than 1,000 ms). Caution is, however, warranted with these interpretations. It is possible that the putative effects of hedonic value or general arousal during the reward learning session may linger into the test session and only gradually become extinct.

It seems more feasible that dopaminergic activity during the test session reflects the prediction error of an “uninformed” learning system. That is, a learning system that simply takes past experiences of stimulus-reward associations into account and is unaffected by what is explicitly known about the associations (e.g., that reward can no longer be earned during the test session). Such a system is akin to what is known as a model-free learning system (O’Doherty, Cockburn, & Pauli, 2017). For a model-free learning system, the transition from training to test session itself is irrelevant; it is the first trials of the test session in which no reward is experienced that matter. Those first trials initially elicit large reward prediction errors, which then gradually reduce with each new reward-absent trial. A correlation between dopaminergic activity and reward prediction error would predict the extinction of dopaminergic activity throughout the course of the test session. This prediction indeed ties in well with Anderson, Kuwabara, et al. (2016), who found differential dopaminergic activity for high and low reward trials only in the first block of their test session. However, similar results are also predicted when assuming that dopamine codes for incentive salience. Here, too, a stimulus that is no longer tied to reward eventually loses its incentive salience. This loss is probably preceded by a gradual decline in dopaminergic activity initiating the regression of the changed neuronal representation. The ambiguity concerning the functional role of dopamine^1^ in reward-based selection history underscores the importance of future studies. Attentional selection studies that use separate training and test sessions in order to disentangle the learning process from the influence of reward-based selection history may prove fruitful in this regard. Certainly, concerns that dopamine is not only reflecting reward learning but also other processes relevant in these type of tasks may still remain.

## Other forms of history effects

In the previous section, we discussed the impact of reward on attentional selection. We specifically discussed evidence for a strong influence of past episodes of rewarded selection on current attentional selection that acts above and beyond known top-down and bottom-up processes. In this section, we briefly discuss evidence from lines of research not concerned with reward that also highlight the importance of past episodes of selection for current selection. Together with the literature on reward history effects, the findings from these studies point toward a common mechanism of lingering biases due to selection history.

### Priming

As briefly mentioned earlier, it has been known for some time that memory processes are capable of shaping attentional selection. One widely known influence of memory processes on attentional selection is priming, which describes how a stimulus (feature) that has been repeatedly attended in the recent past is more efficiently selected and identified on the current trial (Maljkovic & Nakayama, 1994; Tulving & Schacter, 1990). Priming is well-documented in terms of its low-level facilitatory effect on perceptual processing (Kristjánsson & Campana, 2010). Maljkovic and Nakayama (1994), for example, demonstrated the influence of priming in the context of a search task (see also Hillstrom, 2000; Olivers & Humphreys, 2003). Priming between trials, or intertrial priming, occurred for up to eight successive trials, even when participants were unware of repetitions (Maljkovic & Nakayama, 2000), or when they were informed that the target was unlikely to be same between trials (Maljkovic & Nakayama, 1994).

In a critical evaluation of the existing literature regarding feature-based attention, Theeuwes (2013) argued that the role of intertrial priming is often underestimated and its influence on attentional selection is often erroneously attributed to top-down volitional control. In studies on feature-based attention, preparation for a stimulus feature typically requires selective attention to that feature, which then enhances the mandatory processing of it across the visual field. Consequently, the target stimulus that one has prepared for is more easily selected. Even though this effect is typically explained in terms of top-down preparation, it can equally well be explained in terms of priming. When preparing for an upcoming stimulus, attending and processing that stimulus is required to obtain a feature-based attention effect. Therefore, instead of assuming volitional top-down control, the effect may instead be the result of (history-based) lingering biases of selection.

In a similar vein, one can explain the idea of contingent capture (e.g., Folk et al., 1992), which states that selection toward a particular stimulus feature critically depends, at any given time, on the perceptual goals held by the observer. Even though the contingent capture notion has been taken as evidence of top-down attentional control, one aspect in the design of such studies has been greatly overlooked. Crucially, in all contingent capture-like experiments, observers always search for a particular target feature throughout a whole block of trials. In other words, the attentional set is always fixed over a block of trials, which gives rise to strong intertrial priming. If one switches the attentional set randomly from trial to trial (e.g., look for color, or look for an onset) and informs observers to prepare for one of the other attentional set, intertrial priming can be prevented. Importantly, though, contingent capture is then no longer found. Belopolsky and colleagues (Belopolsky, Schreij, & Theeuwes, 2010), for instance, showed that when the attentional set switches randomly from trial to trial, attentional capture by physically salient features (task-relevant and task-irrelevant) cannot be prevented by the observer’s perceptual goals. In other words, the classic contingent capture effects can be easily explained by assuming that selection history drives the effect in the sense that selection is efficient if, and only if, what is selected on the current trial is the same as on the previous trial.

### Statistical display properties

It is well known that contextual information containing invariant properties of the visual environment can bias visual attention. For example, Chun and Jiang (1998) demonstrated the importance of the context in which the selected stimulus is embedded (see also Chun, 2000). They showed that when targets appeared in contextual configurations in which they had been selected before, they were more quickly selected than when they appeared in configurations in which they had never been selected—even though participants seemed to be unaware of the contextual relationships (but see Smyth & Shanks, 2008). Similarly, Wang and Theeuwes (in press) showed how statistical regularities regarding the distractor location can bias visual attention. Using the additional singleton task, participants searched for a salient shape singleton while ignoring a color distractor singleton. Importantly, the color distractor singleton was systematically more often presented in one location than in all other locations. For this high-probability location, Wang and Theeuwes found a reduction in the amount of attentional capture by distractors and a reduction in the efficiency of selecting the target when it happened to appear at this location. Crucially, most participants were not aware of the statistical regularities even though search was biased away from these high-probability distractor locations.

Converging evidence comes from yet another study: Zhao et al. (Zhao, Al-Aidroos, & Turk-Browne, 2013) demonstrated that attention can be biased toward temporal regularities. In one experiment, streams of different abstract shapes were shown at four different locations. These streams were occasionally interrupted by the presentation of a visual search display that contained a target and three distractors in the four stream locations. Importantly, while three streams presented the abstract shapes in random order, one stream contained temporal regularities by presenting the abstract shapes in triplets (i.e., groups of three shapes) with a fixed presentation order. Participants were found to respond quicker when the target happened to be at the stream location that contained temporal regularities relative to when it appeared at the location of a random stream. This effect was observed although the vast majority of the participants were unaware of the temporal regularities. Thus, attention was biased toward the temporal regularities even though they were nonpredictive of the target location, its timing and its identity, and even though participants were unaware of them. Zhao et al. suggested a closed loop of learning these statistical regularities. According to this notion, initial attentional focus is broad so that a bit of each stream is learned. Once visual input matches with was has been learned, attention is drawn toward the spatial location of the regular information. This bias, in turn, prioritizes the spatial location for subsequent episodes of selection effectively reinforcing the learning process.

In short, these studies demonstrate that recent attentional deployments (i.e., selection history) elicit lingering selection biases toward likely target locations and away from likely distractor locations that act above and beyond the influence of top-down and bottom-up control (see also Ferrante et al., 2017).

### Associations with negative valence

Survival in the animal world did not solely depend on efficiency in obtaining reward. It also depended on the immediate detection of potential danger or threat. Numerous studies have demonstrated that this is also reflected in attentional control and suggested that neuronal activity associated with threat- or fear-related stimuli is obligatory (Pessoa, McKenna, Gutierrez, & Ungerleider, 2002; Vuilleumier, Armony, Driver, & Dolan, 2001; Whalen et al., 1998). One of the most powerful examples of these lingering biases of attention is seen in AB tasks in which combat-exposed veterans with posttraumatic stress disorder showed a strong AB for combat images relative to healthy controls (Olatunji, Armstrong, McHugo, & Zald, 2013; cf. Smith, Most, Newsome, & Zald, 2006; for a review, see McHugo, Olatunji, & Zald, 2013). In this sense, the history of being exposed to combat images has an enduring effect on attention. Corroborating evidence comes from fear-conditioning studies which show that fear-associated stimuli strongly bias spatial attention (Mulckhuyse & Dalmaijer, 2016; Wang, Yu, & Zhou, 2013). The basic approach of these studies is to associate a neutral stimulus with the delivery of an electric shock during a fear-conditioning procedure. As a consequence of this procedure, the fear-conditioned stimulus captures attention more strongly than equally salient nonconditioned stimuli (Schmidt, Belopolsky, & Theeuwes, 2015a, b) and biases attention such that the efficacy of sensory processing is enhanced (Preciado, Munneke, & Theeuwes, 2017). Other studies showed that after the association with an aversive event, initially neutral cues caused both facilitated attentional engagement (Koster, Crombez, van Damme, Verschuere, & de Houwer, 2004) and delayed disengagement (Preciado et al., 2017; van Damme, Crombez, & Notebaert, 2008).

Even though these studies demonstrated a strong bias in selecting threat-signaling stimuli, it should be realized that during many, if not all, of these experiments, participants were instructed or encouraged to attend the threat-related stimulus either because it was part of the task (e.g., Koster et al., 2004) or because it was part of the pre-experimental conditioning session (e.g., Mulckhuyse, Crombez, & Van der Stigchel, 2013; Notebaert, Crombez, van Damme, de Houwer, & Theeuwes, 2011). Similarly, the threat-signaling stimulus may have been attended because its physical salience was not carefully controlled (e.g., Öhman, Lundqvist, & Esteves, 2001; Schmidt et al., 2015a) or because it was not associated with any costs for task performance or any influence on the aversive experience (e.g., Schmidt et al., 2015b).

On these grounds, a recent study by Nissens et al. (Nissens, Failing, & Theeuwes, 2016) tested the notion of automatic capture by threat-signaling stimuli in an even more rigorous fashion. The experimental paradigm of their study was analogous to how they investigated the influence of reward on oculomotor behavior while controlling for other top-down and bottom-up processes (cf. Failing et al., 2015). In other words, participants had to make a saccade to a shape singleton while the color of a nonsalient distractor somewhere in the visual field signaled either the possibility of receiving a shock or safety. Critically, attending the threat-signaling stimulus was strongly discouraged: the threat-signaling stimulus was never the target, was never physically salient, and attending it could become harmful because only if participants would make a saccade toward the threat-signaling stimulus could they receive a shock. Participants were explicitly told that if their response was quick enough, then they could avoid receiving a shock. Therefore, if anything, to avoid receiving (more) shocks, the best available strategy was to never attend the threat-signaling stimulus. Nissens et al. observed oculomotor capture by the threat-signaling stimulus even under these aggravated conditions. Strikingly similar to the effect of reward (Failing et al., 2015), the influence of the threat-signaling stimulus was observed early during the selection process as only early fast saccades were prone to oculomotor capture.

In short, there is strong evidence for what has been proposed by major theories on emotion processing (Mathews & Mackintosh, 1998; Öhman & Mineka, 2001): stimuli associated with negative valence have a mandatory and involuntary effect on the allocation of covert and overt attention, which cannot be fully controlled in a top-down effortful fashion. The similarities between the studies concerned with reward and those concerned with negative valence suggest that both factors may influence attentional selection via an, at least partially, overlapping mechanism (for a similar claim, see Belopolsky, 2015; Schmidt et al., 2015b).

## A common mechanism

Many phenomena in attentional control can be explained by adhering to the dichotomy of top-down and bottom-up processes. And yet, we argue that there is by now overwhelming evidence that this dichotomy alone does not stand the test of the empirical evidence because a host of selection biases cannot be explained by either top-down or bottom-up processes. In line with Awh et al. (2012), we therefore propose a third category of bias, incorporating selection history as a vital factor in attentional selection. This third category summarizes processes shaped by past episodes of attentional selection that affect ongoing selection above and beyond competing or concurring top-down and bottom-up processes. Although different types of selection history were discussed throughout this review, from selection history induced by priming or other statistical regularities to selection history based on rewarding or otherwise emotional experiences, we argue that all of them are ultimately relying on the same mechanism. Different factors, such as reward or threat, function as a catalyst or synergist to the learning process. In the following section, we will provide a discussion of the characteristics of the selection history account predominantly using examples of reward-based selection history.

### Selection priority due to (reward-based) selection history

Reward is critically involved in cognitive control, and yet there is mounting evidence for an influence extending beyond the instigation of strategic attentional control. Reward affects attentional selection in a way that cannot readily be explained in terms of active top-down control, as stimuli associated with reward have been shown to disrupt performance even when known strategies to avoid interference were available and better for obtaining reward. Plenty of studies have now demonstrated that this influence of reward cannot be explained in terms of bottom-up processes: reward affects attentional selection even if physical salience of the stimuli involved plays no role. Attention is therefore involuntarily drawn toward stimuli that are or have been associated with reward, irrespective of concurrently active top-down settings or bottom-up saliency signals from the environment.

More generally, recent findings give rise to the idea that reward changes the representation of a stimulus through (repetitive) association. Such an association impinges upon stimulus representations on the level of simple features as well as more complex set of perceptual features, such as objects or scenes. The idea of a changed stimulus representation due to reward history is akin to how Berridge and Robinson proposed “incentive salience” to come about (e.g., Berridge, 2007; Berridge & Robinson, 1998; for a similar theory grounded in the field of associative learning, see the “predictiveness principle” in Le Pelley et al., 2016, or Mackintosh, 1975). According to the incentive salience hypothesis, a theory grounded in research on addiction, incentive salience are the two psychological processes of “wanting” and “liking” associated with a stimulus that reliably predicts reward. Incentive salience transforms an otherwise neutral or intrinsically insignificant stimulus into a stimulus that grabs attention because it signals a wanted outcome. This is reminiscent of a facilitatory priming mechanism driven by reward that renders a reward-associated stimulus more salient to the visual system.

Neuroimaging studies have indeed provided substantial evidence for a change in the neuronal representation of a reward-associated stimulus. These changes are reflected in modulations of neuronal firing such that neuronal activity in response to stimuli imbued with reward history is elevated along the visual hierarchy as early as (extra)striate cortex (e.g., Anderson, 2016b; Anderson, Leal, et al., 2014; Hickey et al., 2010a; Seitz et al., 2009; Serences, 2008; Serences & Saproo, 2010). For example, the fMRI study by Hickey and Peelen (2015) demonstrated that the pattern activity of distractor objects associated with reward was suppressed in the OSC relative to their pattern activity before being associated with reward. Moreover, Hickey and Peelen found larger performance costs due to the reward-associated distractor in participants who showed less suppression of that distractor object in OSC. Findings such as these support the idea that a stimulus associated with reward gains incentive salience, changing its neuronal representation such that the suppression of its representation is necessary in order to prevent it from capturing attention. This idea also dovetails with earlier findings on intertrial reward priming, in which greater activation of a reward-associated stimulus, indexed by a larger N2pc, was quickly followed by a suppression in the visual response to it (Hickey et al., 2010a; see also Qi et al., 2013; Feldmann-Wüstefeld, Brandhofer, & Schubö, 2016).

In terms of attentional control, the concept of a changed stimulus representation connects well with the idea of reward-based selection history (Awh et al., 2012). Through repeated reward-driven attentional selection, a stimulus or its spatial location gains priority in subsequent selection, making it easier and faster to search for it when it is the search target. Conversely, though, such a stimulus or stimulus location also becomes much harder to ignore when it is a distractor presented simultaneously with the target. The opposite also holds: a stimulus for whose suppression one is repeatedly rewarded gains “negative” priority. That is, under specific conditions,^2^ selection priority for a stimulus is reduced below baseline, and that stimulus is more easily ignored when it is a distractor presented simultaneously with the target but more difficult to search for when it is the target.

It is unlikely that this mechanism is unique to reward-based selection history. In fact, we have discussed evidence that similar effects are observed for stimuli that are prioritized in selection due to past selection episodes that were not based on reward per se. These similar effects do not necessarily entail emotional affect, such as fear or threat, but can be the “barest” type of selection history—the repeated selection of an otherwise neutral stimulus or stimulus location. Indeed, not only affective stimuli can become powerful distractors when presented simultaneously with the target but also otherwise inconspicuous stimuli or stimulus locations can come to prime selection based on whether they had been repeatedly selected in the past. Similarly, stimulus locations that often needed to be suppressed in the past continue to be more easily ignored when harboring a distractor and, conversely, searching for a target near the previously suppressed location is far more difficult. Finding that not only selection history based on reward but also on other factors affects ongoing selection suggests that transforming an otherwise inconspicuous stimulus into a stimulus that stands out and demands or repels attention as a consequence of its selection history is a more general property of the brain. We therefore argue that reward constitutes but one type of selection history bias, and each type alike has the ability to change the neuronal representation of a stimulus or stimulus location imbued with it. In this sense, we also argue that reward-based lingering biases due to selection history are in essence not different from other lingering selection history biases (but see Anderson, Chiu, DiBartolo, & Leal, 2017, who argue that lingering reward-based selection biases are fundamentally different than any other nonreward learning effect).

### Modulations on the priority map

**Fig. 1.**
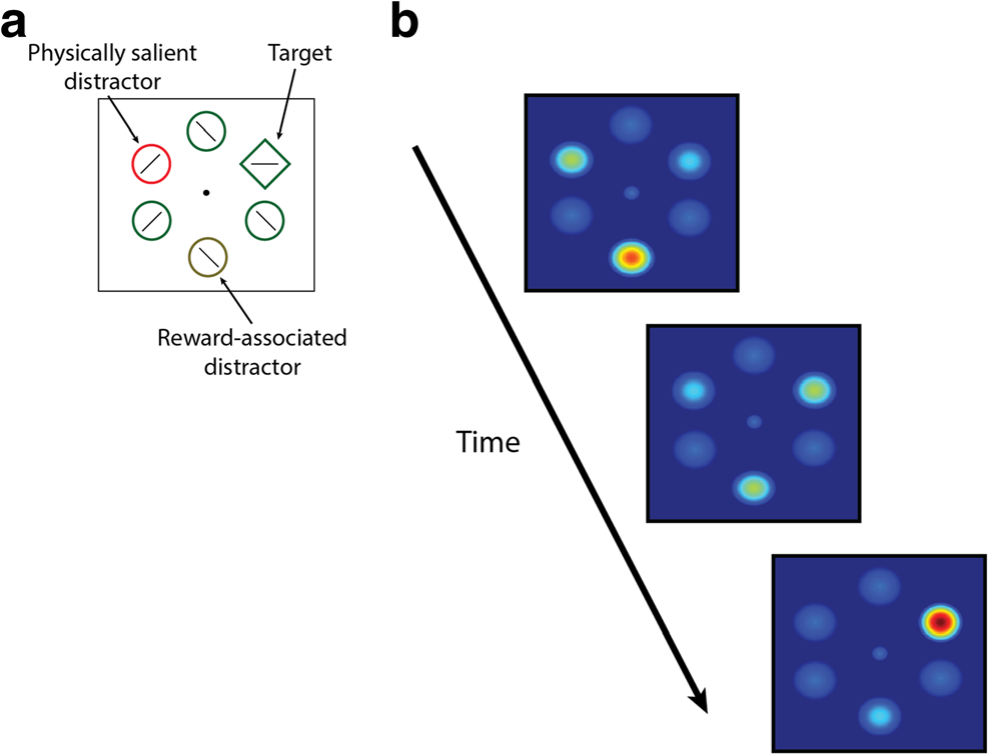
**a** Visual stimuli used in a typical search task. **b** Representation of theintegrated priority map that develops after the onset of the search display, as shown in Fig. 1a.“Warmer” colors represent higher selection priority and thus increased likelihood of attentional selection.Priority signal for attentional selection at each location changes as a function of time: early responsesare more likely to be biased toward spatial locations prioritized by physical salience or reward-basedselection history (priority map on the top), while late responses are more likely to be biased toward task-relevantlocations (priority map on the bottom)

As outlined in the introduction, when adhering to the notion of biased competition, attentional selection can be understood as the process that resolves the intrinsic competition for neural representation (e.g., Desimone & Duncan, 1995). Within this framework, it is presumed that initial competition is dominated by the physical salience of a given stimulus, suppressing neuronal activity of other, less salient stimuli. Later in time, competition can be biased by top-down control—either by prioritizing a spatial location (Moran & Desimone, 1985) or the perceptual feature(s) summarized in an “attentional template” (Chelazzi, Duncan, Miller, & Desimone, 1998; Reynolds, Chelazzi, & Desimone, 1999). The influence of top-down and bottom-up biases are traditionally represented within an integrated priority map of spatial attention. The extension we propose to this model is that the activity within the priority map is also shaped by previous selection experiences (i.e., selection history). Here, we have provided a sketch of factors that drive selection history. We argue that selection history generates a priority signal, much like bottom-up and top-down signals, which is integrated with the other signals within the integrated priority map (Fig. 1). The activity within this map determines in an all-or-none fashion which stimulus is prioritized at any given time. Changes in the neuronal representation of a stimulus location or stimulus feature(s) due to selection history are reflected in changes within the integrated priority map.

In the case of reward, it seems that although reward-based selection history biases competition above and beyond bottom-up and top-down processes, much of the evidence to date suggests that it does so in a similar way as physical salience. This is corroborated by behavioral and neuronal evidence showing that reward is interrelated with the physical salience of a stimulus (e.g., Wang et al., 2013), creates plastic changes in stimulus representations (e.g., Hickey et al., 2010a; Hickey & Peelen, 2015), and affects selection as early as traditional manipulations of physical salience (e.g., Bucker, Silvis, et al., 2015; Failing et al., 2015). Thus, in light of the current evidence, reward learning induces plastic changes in stimulus representations, which are evident as early in visual hierarchy as the (extra)striate visual cortex. By modulating neuronal firing in response to the reward-associated stimulus, these changes will bias initial (early) competition. The relative rapid-acting nature of the reward-based selection history bias may also explain why it oftentimes escapes top-down attentional control and explicit awareness (cf. Theeuwes, 2010). Changes in the stimulus representation during reward learning result in stronger activity for that stimulus on the priority map (i.e., make selection more likely), which reinforces the learning process and thus creates a bias that persists for a prolonged period of time.

Although its specific role remains unclear, there is evidence that the dopaminergic system plays an important role in reward learning and therefore in mediating the plastic changes on the priority map. Since dopaminergic connections are widespread, projecting to brain regions that themselves project activity along the entire visual hierarchy, it is likely that dopaminergic activity during learning is reflected at multiple levels of the visual system. It is therefore conceivable that reward affects the representation of different attributes of a stimulus in different areas of the brain. It may thus also not be surprising to observe the influence of reward on multiple sites within the brain areas that have been implied to represent the priority map. In line with this notion, reward-related activity has been observed in areas consistently associated with the attentional network and believed to represent a form of priority map (e.g., Balan & Gottlieb, 2006; Bisley & Goldberg, 2010; Corbetta & Shulman, 2002; Gottlieb, Kusunoki, & Goldberg, 1998; Li, 2002; Mazer & Gallant, 2003), most notably area LIP/IPS, frontal eye field (FEF), and the SC (e.g., Anderson, Leal, et al., 2014; Dorris & Glimcher, 2004; Ikeda & Hikosaka, 2003; Kobayashi, Lauwereyns, Koizumi, Sakagami, & Hikosaka, 2002; Louie, Grattan, & Glimcher, 2011; Platt & Glimcher, 1999; Peck et al., 2009; Sugrue, Corrado, & Newsome, 2004; Weldon, Patterson, Colligan, Nemeth, & Rizio, 2008; Yasuda & Hikosaka, 2015).

With respect to changed neuronal representations due to selection history effects not based on reward, there is evidence that an equally pivotal role of dopamine exists for affective associations. Indeed, dopamine has been implicated in the learning of affective associations, such as fear-associated stimuli (for a review, see, e.g., Johansen, Cain, Ostroff, & LeDoux, 2011). However, it should be noted that the tasks typically used to study the role of dopamine in the context of affective associations are often not designed to control for factors influencing (selectivity of) attention. Much less is known about what mediates changes in neuronal representations in, for instance, priming. A recent model on intertrial priming, however, suggested that target facilitation effects due to priming may be related to a form of intrinsic reward for successful target selection (Kruijne & Meeter, 2017). This account is supported by observations that reward intertrial priming affects predominantly target representations (e.g., Hickey, Chelazzi, & Theeuwes, 2011; Kristjánsson et al., 2010). Given these findings, we can nonetheless only speculate that, in general, dopamine plays an important role in how selection history comes about and that its role is thus not specific to reward-based selection history.

### Interactions within the priority map

As noted, top-down, bottom-up, and history-driven attentional control signals all reside at the priority map, and at any moment in time interactions between these signals determine which location is selected. The rise of one signal may lead to the inhibition of the other signals, along the lines of biased competition (Desimone & Duncan, 1995) and competitive integration models (Godijn & Theeuwes, 2002; Meeter, Van der Stigchel, & Theeuwes, 2010). It is likely, that salience and history-based effects affect selection early in time while top-down processes dominate selection later in time (see also Hickey, van Zoest, & Theeuwes, 2010c; van Zoest et al., 2004). Even though it is evident that the signals of these three control structures affect each other, it is unclear whether the effects are additive or whether they interact. It is, for example, possible that a nonsalient stimulus that is associated with reward becomes subjectively as salient as a singleton that stands out from its environment due to its physical properties. Also, several studies have already shown that even a strong top-down set to select only the task-relevant stimulus cannot overcome the capturing effects of a salient stimulus (Theeuwes, 1991b, 1992, 1994), a stimulus that is primed (Theeuwes, Reimann, & Mortier, 2006; Theeuwes & van der Burg, 2011), or a stimulus associated with reward (Anderson et al., 2011). In eye-movement studies, fast saccades initiated soon after stimulus onset are more likely to be directed toward the salient stimulus (Theeuwes et al., 1998, 1999; van Zoest et al., 2004) or the stimulus associated with reward (Failing et al., 2015; Pearson et al., 2016) or threat (Nissens et al., 2016). As saccade latency increases, however, more and more saccades are directed toward the target stimulus, signifying that it takes time for top-down control to take effect. Analogous findings are obtained when tracking covert attention with electrophysiological markers such as the N2pc (e.g., Hickey, McDonald, & Theeuwes, 2006; Hickey et al., 2010a).

Previous studies have shown that when attention is highly focused on a location in space, abrupt onset distractors presented elsewhere in the visual field cease to capture attention (Theeuwes, 1991a; Yantis & Jonides, 1990), which demonstrates that focusing spatial attention may be one of the few top-down sets powerful enough to overcome distraction (see also Belopolsky & Theeuwes, 2010). However, recent studies showed that this is not the case for reward-signaling or previously reward-associated stimuli (e.g., MacLean, Diaz, & Giesbrecht, 2016; Munneke, Belopolsky, & Theeuwes, 2016; Munneke, Hoppenbrouwers, & Theeuwes, 2015; Wang et al., 2015). For example, Munneke et al. (2016) found that abrupt onset distractors only broke through the focus of spatial attention when they were associated with reward. When they were not associated with reward, these distractors could simply be ignored, which indicates that physically salient stimuli associated with reward are more powerful in capturing attention than stimuli that are only physically salient.

According to classic accounts of object-based attention, irrelevant elements of an attended object will automatically attract attention (e.g., Kahnemann & Henik, 1981). And yet a series of studies has suggested that reward leads to the complete abolition of object-based attentional guidance (Lee & Shomstein, 2013; Shomstein & Johnson, 2013). Using reward in an object-based cueing paradigm (Egly, Driver, & Rafal, 1994), Shomstein et al. replicated the traditional finding that invalidly cued targets within the cued object are identified faster than invalidly cued targets at an equidistant uncued object. However, this was only the case when there was no reward or when reward was given randomly. In conditions with specific reward schedules, attention spread to the invalidly cued target location within the cued object when it received larger reward than the invalidly cued target location in the equidistant uncued object. Importantly, however, attention no longer spread to the invalidly cued target location within the cued object when the invalidly cue target locations in the equidistant uncued objects received larger reward. Under these circumstances, attention was drawn toward the uncued object.

While these studies demonstrated interactive effects of reward and classic bottom-up processes that drive attentional selection, others provided some evidence for interactive effects of top-down attentional control and reward. For instance, Stankevich and Geng (2015) used a cueing paradigm to investigate whether changes in top-down knowledge about reward-associations leads to immediate changes in attentional prioritization toward them. They found that when participants received explicit instructions about a reversal of the stimulus-reward associations, such that a previously nonrewarded stimulus feature was now associated with reward and vice versa, the direction of the attentional bias did not change immediately. Instead, the attentional bias toward the feature associated with reward changed gradually (for comparable findings in the context of a task where reward-associated stimuli are never task-relevant, see Failing & Theeuwes, 2017). Moreover, adaptation to the original stimulus-reward mapping (i.e., from the very first block) occurred faster than the adaptation to a mapping that was unlike the original mapping. In yet another study, MacLean and Giesbrecht (2015) investigated whether the influence of top-down control can go as far as to modulate early perceptual effects. They observed that the P1 amplitude was increased in response to a stimulus previously associated with high reward, but only when a task-relevant item appeared at the location of that stimulus. Moreover, this effect disappeared when the stimulus previously associated with reward appeared outside of the task-relevant space. It was concluded that top-down task-relevance moderates early perceptual effects of reward-based selection history (but see Hickey et al., 2010a). However, it should be noted that reward training was 7 days prior to the test session. This may indicate that the influence reward-based selection history was strongly diminished such that it could only tip the balance of selection in favor of the stimulus feature previously associated with high reward when task-relevance guided spatial attention toward that feature. Even so, these studies suggest an interactive relationship of top-down control and reward such that top-down biases can affect the impact of reward-based selection history under certain circumstances.

As previously discussed, selection biases are often obtained through a learning phase in which particular stimuli are associated with high or low reward outcomes. During this training phase top-down and bottom-up selection biases also play a crucial role. In order to learn a particular selection history bias, the stimuli involved must receive some attentionally prioritized processing either because they are task-relevant (e.g., Anderson et al., 2011; Theeuwes & Belopolsky, 2012) or stand out from the environment through their physical properties (e.g., Hickey et al., 2010a; Le Pelley et al., 2016). In the former case, top-down processes play a crucial role in acquiring selection biases, while in the latter case, bottom-up processes drive the acquisition of history-based selection biases. Recent attempts have shown that under some circumstances one may obtain attention biases without the need for specific attentional prioritization by means of top-down task-relevance or bottom-up salience (e.g., Bucker & Theeuwes, 2017b; Failing et al., 2015; Failing & Theeuwes, 2017).

### Outstanding questions and future directions

In this final section, we outline several outstanding questions regarding the relationship of reward-based selection history and attention that have not already been explicitly addressed in this review. Although these questions are predominantly from a reward-based selection history point of view, we believe that many of the issues raised extend to the other types of selection history biases as well.

#### Attentional suppression

At least one study demonstrated that “attentional suppression responses” can be learned in a way akin to instrumental conditioning (Della Libera & Chelazzi, 2009). That is, when attentional suppression of a specific stimulus was rewarded during training, that stimulus was also more efficiently suppressed during a test session. However, other studies observed no such learning under conditions in which attentional suppression would have been the appropriate response that also would have led to more reward payout. These studies instead demonstrated that a reward-signaling stimulus demanded attention irrespective of it being detrimental to task performance and reward payout (e.g., Le Pelley et al., 2015; Failing et al., 2015; Failing & Theeuwes, 2017). A similar discrepancy with respect to learning attentional suppression appears to exist regarding selection history effects without affective modulations. For instance, while intertrial priming studies typically show that a recently selected stimulus feature is more likely to be selected again, even if it is a distractor whose suppression was and is the appropriate response (Pinto et al., 2005), other studies suggest that one can learn to suppress a specific stimulus location if it is the appropriate response (Wang & Theeuwes, 2017; Ferrante et al., 2017). With respect to the studies concerned with selection biases unrelated to reward, it may be argued that locations within the priority map can be suppressed (Theeuwes, 2013), while specific features within this map cannot. However, the recently proposed “signal suppression hypothesis” (Sawaki & Luck, 2010) states that features of an irrelevant distractor that generates a strong bottom-up signal can be actively suppressed in a top-down way such that the distractor no longer captures attention (see also Gaspelin, Leonard, & Luck, 2015). The explanation for why some reward studies find suppression while others find prioritization is even less clear. Even though they highlight the difference in instrumental and Pavlovian type of learning, the conditions that determine which of the two drives the acquisition of the selection history bias remain unclear.

#### Modeling the influence of reward-based selection history on eye movements

It is currently unclear how existing oculomotor models can best account for some findings regarding reward-based selection history. For instance, Failing and colleagues observed oculomotor capture of stimuli that signal high reward relative to those that signaled low reward but no difference in saccadic latencies (Failing et al., 2015; Pearson et al., 2016; see also Theeuwes & Belopolsky, 2012).^3^ The authors explained this pattern in the context of the competitive integration model (Godijn & Theeuwes, 2002; for a brief summary, see the Priority Maps: An Integration of Top-Down and Bottom-Up Processes section). Since oculomotor capture by reward-signaling but otherwise nonsalient stimuli was observed particularly for the early first saccades, it is likely that the reward signal is already integrated at an early stage of the selection process. Similar to the typical interpretation of oculomotor capture by physically salient stimuli, Failing and colleagues argued that the location of the reward-signaling stimulus is likely to have a high activity on the saccade map as well, which then in turn inhibits activity of other distant locations (e.g., the location of the target) through lateral inhibition. However, if that were to be the case, one would have also expected a difference in saccadic latencies due to stronger inhibition of saccadic activity for the distant target location. Finding no consistent modulation in saccadic latencies due to reward-signaling distractors may therefore suggest that reward simply lowers the threshold, which must be surpassed in order to elicit a saccade in a feature-specific manner (Failing et al., 2015; Pearson et al., 2016). Alternatively, reward may give an independent boost to the saccade program to the distractor’s location without affecting the lateral inhibition of other stimuli (i.e., the target; Belopolsky, 2015). More generally speaking, reward, unlike physical salience, may not affect lateral inhibitions at all. Whatever the mechanism may be, further research will be necessary to establish how existing oculomotor models can best account for the empirical data from oculomotor studies on reward-based selection history.

#### Generalization of reward-based selection history

In most studies reviewed, the specific task and task set during learning of the reward associations is very similar to the task during testing. In that sense, it may not be surprising that a stimulus that is trained to generate a high reward for fast responding continues to interfere with search for at least the first few hundreds of trials, even when the target is different. Some studies have shown, however, that learned associations keep affecting selection even when the training and test task are very different (e.g., Chelazzi et al., 2014; Jahfari & Theeuwes, 2016; Lee & Shomstein, 2014; Raymond & O’Brien, 2009). Similarly, the stimulus feature associated with reward during learning is typically identical to the reward-associated feature during testing, and the influence of reward-based selection history has been suggested to be highly context-specific (Anderson, 2015). However, there is also evidence that reward generalizes to similar features as the one that has been associated with reward (Failing & Theeuwes, 2015; Anderson, 2017). The extent to which generalization of selection history effects occur across context, tasks, and stimulus features needs to be investigated systematically, as this will provide important boundary conditions.

#### Individual differences in reward-based selection history

The likelihood and strength by which previously reward-associated stimuli affect attentional selection varies substantially between individuals. Several factors that contribute to these variations have already been discovered. For instance, while depressed patients exhibit blunted VDAC (Anderson, Leal, et al., 2014), addicts show increased VDAC (Anderson et al., 2013). Young individuals are more prone to distraction by previously reward-associated stimuli than older individuals (Roper et al., 2014), and males demonstrate greater priority for reward-associated locations than females (Della Libera, Calletti, Eštočinová, Chelazzi, & Santandrea, 2017). Moreover, individual working memory capacity has been shown to be negatively correlated with capture by previously reward-associated stimuli (Anderson et al., 2011; Anderson et al., 2013). Higher impulsivity (Anderson et al., 2011, 2013) as well as stronger behavioral approach drive (Hickey, Chelazzi, and Theeuwes, 2010b) and reward responsiveness (Hickey & Peelen, 2015) as measured by the Barratt Impulsiveness scale (Patton, Stanford, & Barratt, 1995) and BIS/BAS questionnaire (Carver & White, 1994) have been associated with a larger effect of reward on attentional selection, although the contribution of each of these factors has not consistently been reported. Altogether, these findings underscore that the strength of the reward-based selection history bias can vary significantly between individuals as a function of differences in pathologies or traits. Studies that manipulated the state of individuals to observe reward effects (e.g., by depriving them of water: Seitz et al., 2009; or food: Cunningham & Egeth, 2016) suggest that state-dependent fluctuations within a single individual may also affect the expression of reward-based selection history bias. In light of this variety, it seems unlikely that we have a complete understanding of all the factors contributing to the variations in reward-based selection history bias.

#### Brain mechanism of reward-based selection history

Research on reward-based selection history has highlighted some of the key brain areas that have traditionally been linked to reward, such as the basal ganglia, more specifically, the rostral and caudal regions of the caudate nucleus in the striatum, and the VMPFC, which directly receives input from the basal ganglia (e.g., Anderson, Kuwabara, et al., 2016, 2017; Hickey & Peelen, 2015; Hikosaka et al., 2014; Vaidya & Fellows, 2015). Reward-based selection history has also been reflected in activity of brain areas traditionally associated with attention, such as the OSC and IPS (Anderson, Leal, et al., 2014; Hickey & Peelen, 2015; Peck et al., 2009). Although it seems that all pieces of the puzzle are in place, it is unclear how the reward signal is communicated from brain areas that are more concerned with reward (learning) to areas that code for selection priority. Dopamine has been implicated to convey such information (Anderson, Kuwabara, et al., 2016; Anderson, Chiu, et al., 2017) but given the fast-paced dynamics of attentional selection it is unlikely that communication is purely neuromodulatory in nature. Neuronal synchronization has been proposed as a key mechanism in communicating information from one brain area to another (Fries, 2005) and to play a vital role in memory (Fell & Axmacher, 2011) as well as attention (Jensen, Gips, Bergmann, & Bonnefond, 2014). A promising avenue for future research may thus lie in investigating the neuronal synchronization between the respective brain areas and/or circuitries.

Another question relates to how reward-based selection history that is either primarily driven by Pavlovian or instrumental associations is reflected in the brain. A distinction between both has been demonstrated in behavioral studies (Della Libera & Chelazzi, 2009; Le Pelley et al., 2015; see also Chelazzi et al., 2013) but, to date, this difference has not been explicitly addressed by any neuroimaging or electrophysiological study. Thus, it remains unknown whether the acquisition and expression of reward-based selection history driven by Pavlovian or instrumental conditioning occurs via shared or fundamentally different pathways in the brain.

## Conclusions

Decades of research have provided evidence that attentional control is the result of a mixture of top-down and bottom-up processes. In the present article, we argue that this dichotomy falls short in explaining a wide range of phenomena that may be better accounted for by lingering biases of attention due to selection history. The underlying idea is that past attentional selection episodes strongly bias current selection above and beyond top-down and bottom-up processes. This is well-illustrated by the interactions of reward and attention. Not too long ago, it was generally accepted that reward predominantly affects top-down processing. Reward incentives motivate individuals to perform better by promoting cognitive and executive control. Only recently, however, research has demonstrated that reward shapes perceptual as well as attentional processes above and beyond known top-down and bottom-up processes. Reward prioritizes stimuli for covert or overt search and modulates spatial as well as temporal attentional selection. This prioritization is achieved by changes in the neuronal representation of a stimulus imbued with reward-based selection history such that it becomes more pertinent to the visual system. In addition to reward-based history effects, lingering selection biases due to past selection episodes can also be induced by emotion, priming, and statistical regularities.

## Footnotes

1 At this point, it should be once more emphasized that the specific role of dopamine in reward (learning) is still a matter of debate, even outside the field of attention. Specifically, the causal relationship of the dopaminergic response and reward has been a focus of discourse. The interested reader is referred to other reviews discussing the role of dopamine in reward in great detail (e.g., Berridge, 2007; Berridge & Robinson, 1998; Kringelbach & Berridge, 2009; Schultz, 2013, 2016).

2 It should be noted that, as of yet, the specific conditions that determine whether selection priority is reflected in attentional enhancement or attentional suppression are unknown. More specifically, some studies seem to find enhancement when suppression would be expected (see the Outstanding Questions and Future Directions section for more detail).

3 Several studies found an effect of reward on saccadic latencies (Le Pelley et al., 2015; Pearson et al., 2015). However, it should be noted that in these studies, reward was *always* associated with the physically most salient item in the display. As outlined earlier, it is well known that physically salient distractors affect saccadic latencies. Although the salience of each reward-associated stimulus was matched, it is thus still possible that the differences found in saccadic latencies were a consequence of an interaction between salience and reward during the learning process. It is therefore difficult to assess in how far reward-based selection history alone affected saccadic latencies in these studies.
